# Left ventricular and atrial function in systemic light chains amyloidosis: an echocardiography and cardiac magnetic resonance comparison

**DOI:** 10.1186/1532-429X-17-S1-P369

**Published:** 2015-02-03

**Authors:** Dania Mohty, Cyrille Boulogne, Thibaud Damy, Julien Magne, Jean François Deux, Victor Aboyans, ARNAUD Jaccard

**Affiliations:** 1 Cardiology, CHU Limoges, Limoges, France; 2 Cardiology, CHU Henri Mondor, Créteil, France; 3 Hematology, CHU Limoges, Limoges, France

## Background

Cardiac involvement in systemic light-chain amyloidosis (AL) is characterized by slightly decreased systolic left ventricular function (LV) function and typically a diastolic dysfunction including left atrial (LA) enlargement. Cardiac magnetic resonance (CMR) is often performed in AL to accurately assess chambers size and function. We aimed to compare features of LV systolic and diastolic function obtained by 2D transthoracic echocardiography (TTE), with morphological and functional myocardial LV (presence of late gadolinium enhancement: LGE) and LA (volume and emptying fraction) parameters as assessed by CMR in a consecutive series of patients with cardiac AL.

## Methods

Thirty-five consecutive patients in sinus rhythm (66±10 years, 59% males) with confirmed systemic AL, underwent TTE and CMR within the same day. LVEF and LV 2D global longitudinal speckle tracking imaging obtained by TTE were stratified according to the presence or not by CMR, of myocardial late enhancement gadolinium (LV-LGE). Left atrial emptying fraction (LAEF) was calculated after assessing the maximal and minimal LA volume (by area/length formula) in CMR using 4 and 2 chambers views and the following formula: Max LAV-Min LAV/ Max LAV

## Results

**Figure 1:**
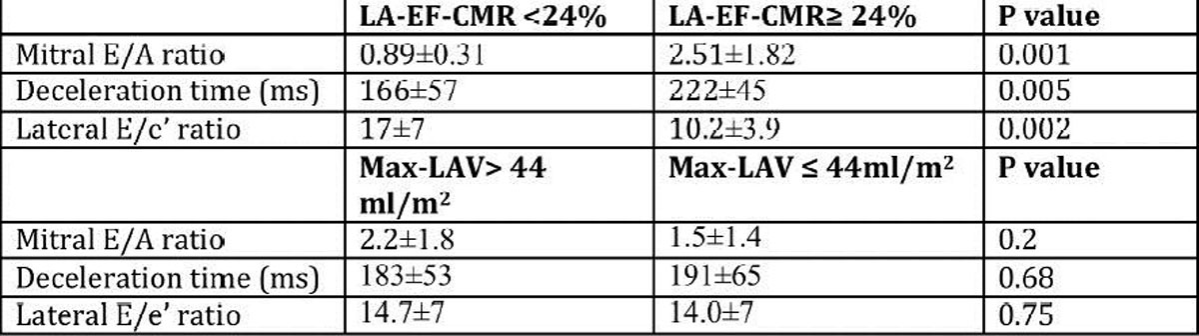


2D- LVEF and global longitudinal speckle tracking imaging were significantly decreased in patients with LV-LGE when compared to those without: 56.5±10.4% vs. 63.5±10.2%, p=0.04 and -10.8±2.8% vs. -16.6±5.02%, p=0.0005 respectively. Diastolic mitral deceleration time and E/A ratio and E/e' at lateral annulus were significantly altered in patients with low LA-EF < 24% median value) vs. those with higher LA-EF whereas, they were not significantly different according to max-LAV by CMR (Figure 1)

## Conclusions

In systemic AL, reduced systolic LV 2D speckle tracking imaging is significantly associated with the presence of LV-LGE while impaired LV filling pressures are rather related to decreased in LA emptying fraction. Multimodality imaging in patients with AL may allow complementary and better assessment of LV hemodynamics. However, larger series are needed to confirm these results.

## Funding

None.

